# How “Sure” Are We About Precision Medicine in IBD?

**DOI:** 10.1002/ueg2.70048

**Published:** 2025-05-19

**Authors:** Florian Tran, Konrad Aden

**Affiliations:** ^1^ Institute of Clinical Molecular Biology University Hospital Schleswig‐Holstein Christian‐Albrechts‐University Kiel Germany; ^2^ Department of Internal Medicine I University Hospital Schleswig‐Holstein Christian‐Albrechts‐University Kiel Germany

The management of Inflammatory Bowel Disease (IBD), particularly Crohn's disease (CD) and ulcerative colitis (UC), has advanced significantly over the past two decades. However, the aspiration for personalized treatment strategies remains a challenge due to the unpredictable nature of disease progression. The early identification of severe disease course is particularly important in CD patients, in which an early therapy escalation into a more aggressive treatment might be able to prevent long‐term tissue damage. Till now different technological approaches and omics‐layers have been exploited to generate prediction tools, incl. microbiome, metabolomics and transcriptomic, on the disease course of IBD such as the need for or the response towards targeted therapies [1]. The **PredictSURE IBD assay** is a blood‐based prognostic test designed to stratify newly diagnosed, treatment‐naïve patients with IBD into high‐risk (IBDhi) and low‐risk (IBDlo) groups. This classification is based on a 17‐gene expression signature derived from CD8+ T cells, which has been shown to correlate with the likelihood of experiencing aggressive disease progression, including the need for treatment escalation and surgery [2]. Recently the utility of this test has been explored in a landmarking trial (PROFILE), in which CD patients were stratified into high‐risk (IBDhi) and low‐risk (IBDlo) groups and subsequently treated with an accelerated step‐up or top‐down approach. This trial failed to provide evidence for the predictive utility of PredictSURE but strongly advocated towards early and aggressive advanced drug therapy intervention in CD patients [3]. In the current issue Alsoud et al. assessed the predictive power of **the assay for the first time in a multinational cohort** of a total of *n* = 166 UC and CD patients [4]. They found that IBDhi UC patients required significantly more treatment escalations and had a shorter time to their first escalation compared to their low‐risk (IBDlo) counterparts. However, the results were less promising for CD patients, where no significant differences in treatment escalations were observed between the biomarker‐defined groups. Importantly this observation is in line with the previous report on the utility of PredictSURE in guiding step‐up versus top‐down therapeutic approaches in CD patients. However, their findings come with a critical caveat: an important aspect of the study is the impact of corticosteroid therapy on the assay's performance. Alsoud et al. report that the biomarker's discrimination ability was unreliable in patients' receiving corticosteroids, with many patients classified as IBDlo requiring treatment escalations. Hence this study also raises several important questions regarding the future design of biomarker for precision medicine. It starts with the simple question why a biomarker is working in UC but not in CD? It also needs further debated to which extent biomarker might be used in clinical practice for the prediction of disease course of an IBD subtype which accuracy is confounded by concomitant steroid use? In that sense it is particularly interesting that CD patients within the PROFILE trial all received an 8‐week course of oral steroids, which might be a reason why the diagnostic accuracy was not reached in the PROFILE trial. After all the presented study provides a vivid example on how to close the current gap between precision medicine in IBD as future dream and our current clinical reality. And it also “painfully” underlines again the critical dogma in IBD therapy: be aware of corticosteroids!

## Conflicts of Interest

The authors declare no conflicts of interest.

## Data Availability

The authors have nothing to report.
